# Application of error level analysis in image spam classification using deep learning model

**DOI:** 10.1371/journal.pone.0291037

**Published:** 2023-12-14

**Authors:** Angom Buboo Singh, Khumanthem Manglem Singh

**Affiliations:** National Institute of Technology, Manipur, India; Mirpur University of Science and Technology, PAKISTAN

## Abstract

Image spam is a type of spam that contains text information inserted in an image file. Traditional classification systems based on feature engineering require manual extraction of certain quantitative and qualitative image features for classification. However, these systems are often not robust to adversarial attacks. In contrast, classification pipelines that use convolutional neural network (CNN) models automatically extract features from images. This approach has been shown to achieve high accuracies even on challenge datasets that are designed to defeat the purpose of classification. We propose a method for improving the performance of CNN models for image spam classification. Our method uses the concept of error level analysis (ELA) as a pre-processing step. ELA is a technique for detecting image tampering by analyzing the error levels of the image pixels. We show that ELA can be used to improve the accuracy of CNN models for image spam classification, even on challenge datasets. Our results demonstrate that the application of ELA as a pre-processing technique in our proposed model can significantly improve the results of the classification tasks on image spam datasets.

## 1. Introduction

Spam mail are becoming more of a security threat [1] than an annoyance. The classification of spam mail is a major challenge faced, as the adversaries are constantly updating the tools and techniques for the creation and dissemination of spam mails. Recent advancement in the area of machine learning [2,3] have proven to be useful in the area of spam classification tasks.

Image spam is a form of spam with embedded text information inside a base image file. The text information is embedded into the image file to evade the common practise of using text filtering in spam classification task. With advanced text embedding techniques [4], extracting the text using OCR is even proving to be difficult. Alternatively, instead of using normal text filtering techniques, image spam filtering is performed using features that are extracted from the input spam images. The accuracies, complexities and performance of such filter depends mainly on the types of image features, extraction method used and classification algorithm adopted.

One commonly used approach in the image spam classification requires manual feature engineering of different type of features and the selection of the most appropriate and useful one. Larger number of features usually results in better accuracies at the cost of computation and therefore many approaches focus on selecting useful features to improve the classification accuracy and reduce the overall computation. However, as the adversaries are using various image processing techniques to make the image spam looks more like non-spam, the manual feature engineering is becoming less accurate in the classification task.

Given that Image spam are usually created by editing or changing part of the base image to include certain textual information and therefore will results in different compression level in the image. These differences in the compression level in the image could be highlighted by applying error level analysis [5] in the input image. Therefore, we proposed to apply error level analysis to the input image in this research as a pre-processing technique and create a more distinct image features, which will improve the result of the spam classification task. One of the drawback of our approach is that error level analysis works only on lossy compression such as JPEG images and other image format such as PNG are not supported. Moreover, to overcome the challenges of the manual feature engineering and selection of useful features, we propose to use Deep Learning techniques [6–10]. Using this approach, the features are automatic extracted from the input image and therefore the accuracy and the complexity is improved. However, one of the issue in using a Deep Learning technique is the requirement of a large datasets for training and high compute to obtain a fine tuned model.

To sum up, our main contribution in the paper are as follows:

- Application of Error Level Analysis as a pre-processing techniques to the input images
- Use of Deep Learning approach for automatic feature extraction
- Fine-tuning of the deep learning model to improve the classification task

The rest of the sections of this work are organized as follows; Section 2 presents the related works. Section 3 contains the dataset description. Section 4 presents the proposed methodologies. Section 5 includes experimental results and discussion. Finally, the conclusion is placed in Section 6.

## 2. Related work

### 2.1 Image spam detection

Optical Character Recognition is widely used to extract textual information from a given image. Such approach is also used in image spam detection by first extracting the textual information from the given image and then applying the different text classification techniques [11–14] for the classification purpose. Textual features such as header, body, BoW, Structure, hyperlinks, attachments, Term Frequency etc [15] are commonly used features in the detection of spam mails. Advanced features such as rank score [16] which is generated by using the linkage information of the image, textual information of the image, and metadata information of the image helps improved the classification accuracy by increasing the relevance of the input image.

Instead of using textual features, many authors have used image features directly from the input spam images for the classification task. However, the resulting performance and the detection accuracy depends on the type and number of image features used. Different author manually generate the image features based on properties and meta data of the image file [17], global features including color and gradient histogram of the image file [18–24], some form of low level image features [25–29], Image texture based features related to run-length matrix, auto-regressive model, co-occurrence matrix, wavelet transform, histogram and gradient [30–32]. Other work uses image features based on Speeded Up Robust Features (SURF) [33] and n-gram feature from the Base64 format of the image file [34]. Different machine learning techniques such as KNN classifier [35], SVM [36–37], are applied for improving the classification task.

Improvement in the classification accuracy has been observed by using various form of CNN [38], Transfer Learning based on Pre-Trained Deep Learning models [39] and new pre-processing techniques such as illumination normalization techniques [40]. These models show very high accuracy even on improved [36] and challenge [37] datasets, which are especially handcrafted by superimposing the spam image on the non-spam images.

Our main contribution in this paper is that, we apply error level analysis to the input spam images, to further enhance the image features and thereby improve the accuracy and performance of the classification tasks and fine-tuned the model to improve the classification accuracy.

### 2.2 Error Level Analysis (ELA)

Some of the image format such as the JPEG is lossy in nature and uses the transform compression such as Discrete Cosine Transform to retain the low frequency components and when image is saved or resaved, some errors are introduced. However, the amount of error introduced by each resave is not linear, and when an image is modified, the 8x8 cells containing the modifications are no longer at the same error level as the rest of the unmodified image.

Error Level Analysis (ELA) [5] is a technique to identify various portions of an image with a varying level of compression ratio. This is achieved by resaving the image at a known error rate, and then computing the difference between the resaved image and the original image as given below.

Error_level=(Px-Py) (1)

where:

*Error_level* is the difference between the original pixel value and the compressed pixel value

*P*_*x*_ is the original pixel value in the image

*P*_*y*_ is the compressed pixel value in the image

Many works in the field of image forensics uses ELA for the identification of tampered area in the input images. The works of [41–44] are based on the above approach of using ELA in image as well as video for forgery detection.

**Fig 1:**
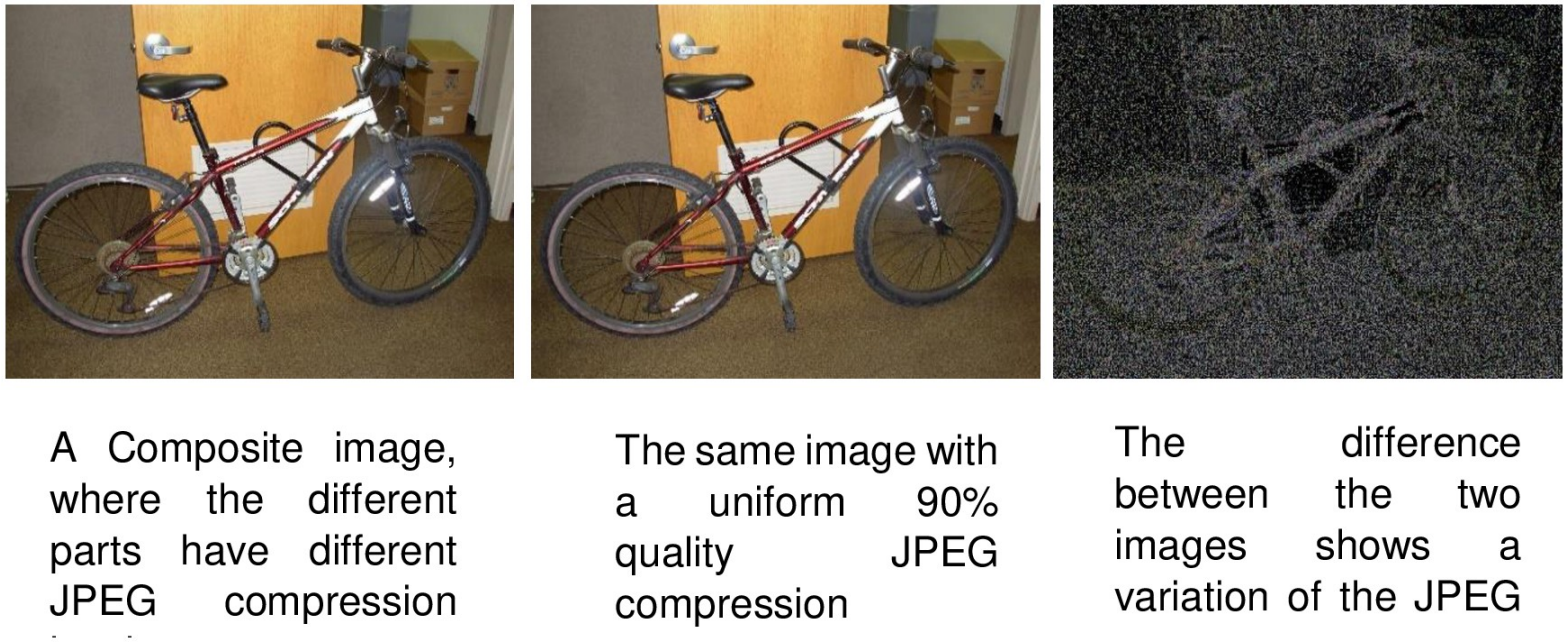
Examples of error level analysis images.

Image spams created by embedding the spam text in ordinary JPEG images usually introduce different level of compression in the embedded text portion. Using ELA, the image features extracted can enhance and improves the accuracy and performance of the detection task. The limitation of applying ELA is that, only lossy compression images such as JPEG format are supported and the result may be affected by the compression level used to generate the ELA. When lower compression level is used, the ELA image may not be able to detect any areas of manipulation. However, if the compression level is too high, then the ELA image may identify false positives. An example of an Error Level Analysis images is shown in Fig 1.

## 3. Material and methods

This section discussed the datasets used in performing the different experiments along with the various deep learning models used.

### 3.1. Image spam datasets used

The details of the datasets used in the experiment are shown in Table 1.

**Table 1:** Datasets Used in the experiment.

Sl No	Dataset Name	Total Spam used	Total Non-Spam used	Remark
1	Dredze [17]	1089	1029	This dataset consist of 2550 non–spam images, 3239 spam image and 9503 Spam Archive Spam images.
2	Image Spam Hunter [18]	920	810	This data set consists of 810 non–spam and 928 spam images.
3	Improved[36]	1029	810	This dataset contains 810 non–spam and 1029 hand crafted Improved spam images.
4	Challenge A [37]	810	810	This dataset consists of 810 non–spam and 810 hand crafted challenge spam dataset.
5	Challenge B [37]	810	810	This dataset consists of 810 non–spam and 810 hand crafted challenge spam dataset.

### 3.2. Convolutional neural network model

A convolutional neural network (CNN) is a type of artificial neural network that is specifically designed for processing data that has a grid-like structure, such as images. CNNs are able to learn to identify patterns in images, and they are often used for tasks such as image classification, object detection, and image segmentation. The basic idea behind a CNN is to use a series of convolutional layers to extract features from an image. A convolutional layer is a type of neural network layer that applies a filter to an image, which helps to identify specific features in the image. The filters are learned during the training process, and they are typically based on the features that are known to be important for the task at hand.

Once the features have been extracted, they are passed to a series of fully connected layers, which are responsible for classifying the image. The fully connected layers learn to map the extracted features to the labels of the different classes of images.

Pre-trained convolutional neural network models, which are trained on a large dataset can be used to save time and efforts by using the transfer learning technique. In our experiment, we choose the Pre-trained CNN model: BiT-M R50x1 [45], a high performing pre-trained model as our base model.

#### 3.2.1 BiT–M R50×1

**Fig 2:**
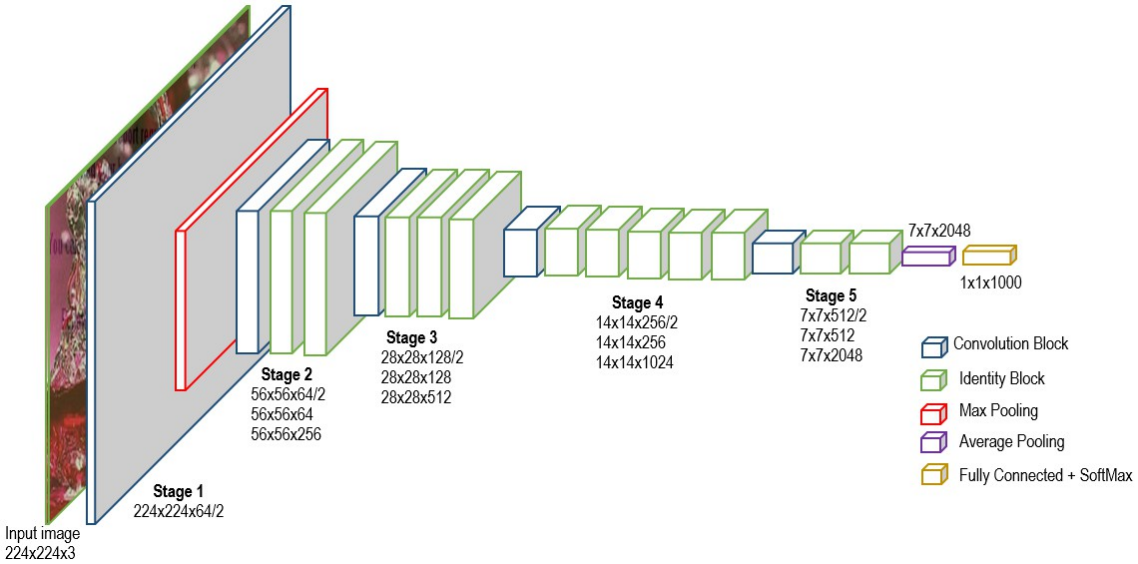
BiT–M R50×1 model architecture, image by author.

**Fig 3:**
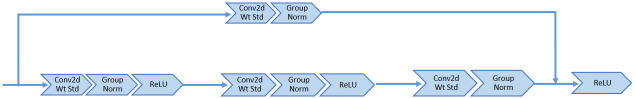
Convolution block, image by author.

**Fig 4:**
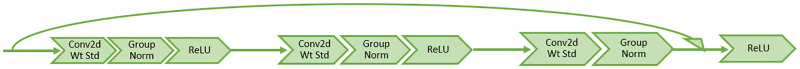
Identity block, image by author.

The BiT-M R50x1 model is a high performance CNN model which is based on ResNet and is shown in Fig 2. The model takes as input a 224×224 colour image and generates a 2048–dimensional features vectors as output and consists a multi-class classifier as head. The model consists of Convolution Blocks shown in Fig 3 and Identity Blocks as given in Fig 4. The convolutional block in BiT-M R50x1 consists of a sequence of convolutional layers, followed by a shortcut connection. The shortcut connection simply adds the input to the output of the convolutional layers. This allows the convolutional block to learn residual connections, which helps to prevent the vanishing gradient problem. These blocks are of different dimensions and pooling are applied to these blocks to further reduce the dimension.

The Big Transfer (BiT) models are a powerful set of pre-trained image models that can be used to achieve excellent performance on a variety of tasks, even with few labeled samples. The models are based on the ResNet 50 architecture and are pre-trained on a large supervised dataset. They are then efficiently tuned for specific target tasks using a technique called transfer learning.

One of the key innovations of the BiT models is the use of group normalization and convolutional core weight normalization. These normalization techniques help to improve the performance of the models by making them more robust to changes in the input data. As a result, the BiT models are able to achieve state-of-the-art performance on a variety of tasks, including image classification, object detection, and image segmentation.

## 4. Proposed classification model

There are three main components in the proposed model:

1. ELA image generator.
2. Feature extractor; and
3. Binary classifier.

**Fig 5:**
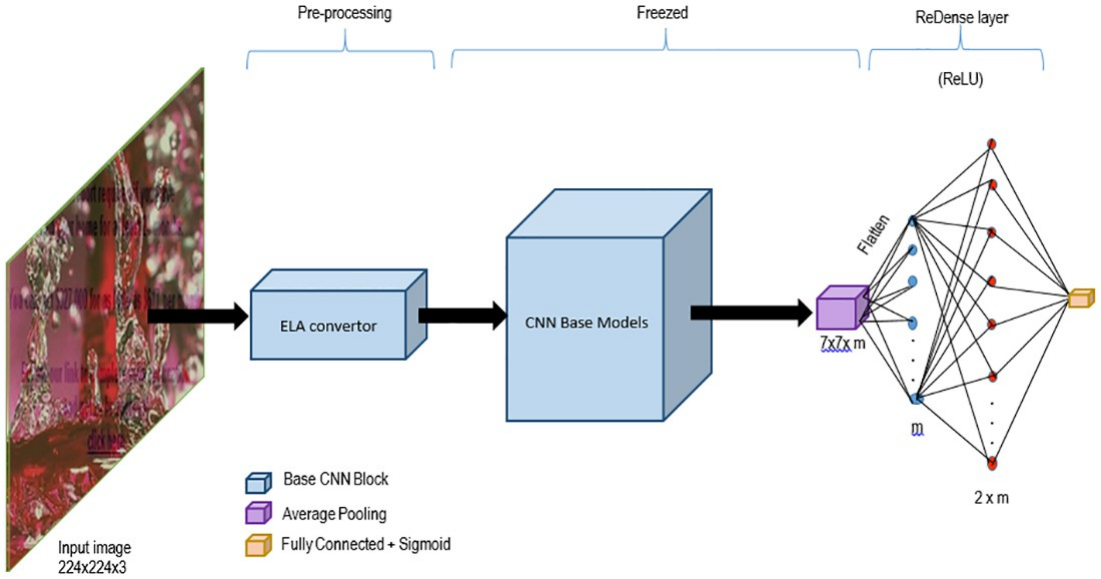
Proposed model, image by author.

The proposed model consisting of the ELA image generator, the base CNN model and the final classification unit is shown in Fig 5.

The ELA image generator is part of the pre-processing module where the pre-processing on the datasets are performed. The main pre-processing carried out are:

- Resizing the input images to 224×224 dimensions,
- Normalizing the image data to values between 0–1.
- Generation of ELA Image by taking the pixel difference of the input image and the compressed input image.

The next module is the Base CNN model which has been strip of its classification head. The output of the ELA image generator is fed into the CNN model to extract the features. This is achieved by first freezing the convolution blocks along with the Identity and the pooling blocks of the Base CNN model. The input images of dimension 224×224×3 is then transformed into a 7×7×2048 through multiple stages consisting various blocks as shown in Fig 2 and finally it is flattened to output a 2048 dimensional feature vector.

Finally, the binary classification module consists of two dense layers as the last layer in the model. The first dense layer is of the dimension 1×1×2*n* ReDense Layer [46], where *n* is the dimension of the flatten vector and uses a Rectified Linear Unit as its activation function and the second dense layer is the output classification layer which uses a Sigmoid activation. The use of these two dense layers provides an improvement in the performance of the detection.

The parameters in the proposed model are shown in Table 2.

**Table 2:** CNN architectures.

Base Model Name	Number of Layers	Output Feature dimension	Number of Parameters
BiT-M R50x1	50	2048	23500352

Fine tuning is done through different values in the network hyper-parameters. The values provided in Table 3, results in the highest accuracies.

**Table 3:** Network hyper-parameters.

Batch-Size	Learning-Rate	No. Epochs	Optimizer	Loss-Function
32	1×10^−3^	25	Stochastic Gradient Descent with Momentum	Sparse-Categorical Loss

## 5. Experiments and result

### 5.1. The experimental frameworks

The following frameworks were used in performing all the experiments:

- Python 3.6 with OpenCV [47].
- Intel workstation with Xeon Quad-Core processor, 32 GB RAM
- MS Windows 10 Pro 64bit,
- Nvidia Quatro-4000 GPU, 4GB VRAM
- Keras deep learning framework [48].

### 5.2. Performance measures

The performance measure is calculated using different evaluation indicators. Some of the measures are given below:

$$Accuracy=\frac{TP+TN}{TP+TN+FP+FN}$$ (2)

$$Recall=\frac{TP}{TP+FN}$$ (3)

$$Precision=\frac{TP}{TP+FP}$$ (4)

$$F1-score=\frac{2\times \left(Precision\times Recall\right)}{Precision+Recall}$$ (5)

Where,

FP: The number of misclassified legitimate emails;

FN: The number of misclassified Spam;

TP: The number of correctly classified Spam;

TN: The number of correctly classified legitimate emails.

The confusion matrix can be used to define the performance of the classification algorithm and is given below in Table 4.

**Table 4:** Confusion matrix.

Predicted	Actual	
	Spam	Non spam
Spam	TP	FN
Non Spam	FP	TN

### 5.3. Results

**Fig 6:**
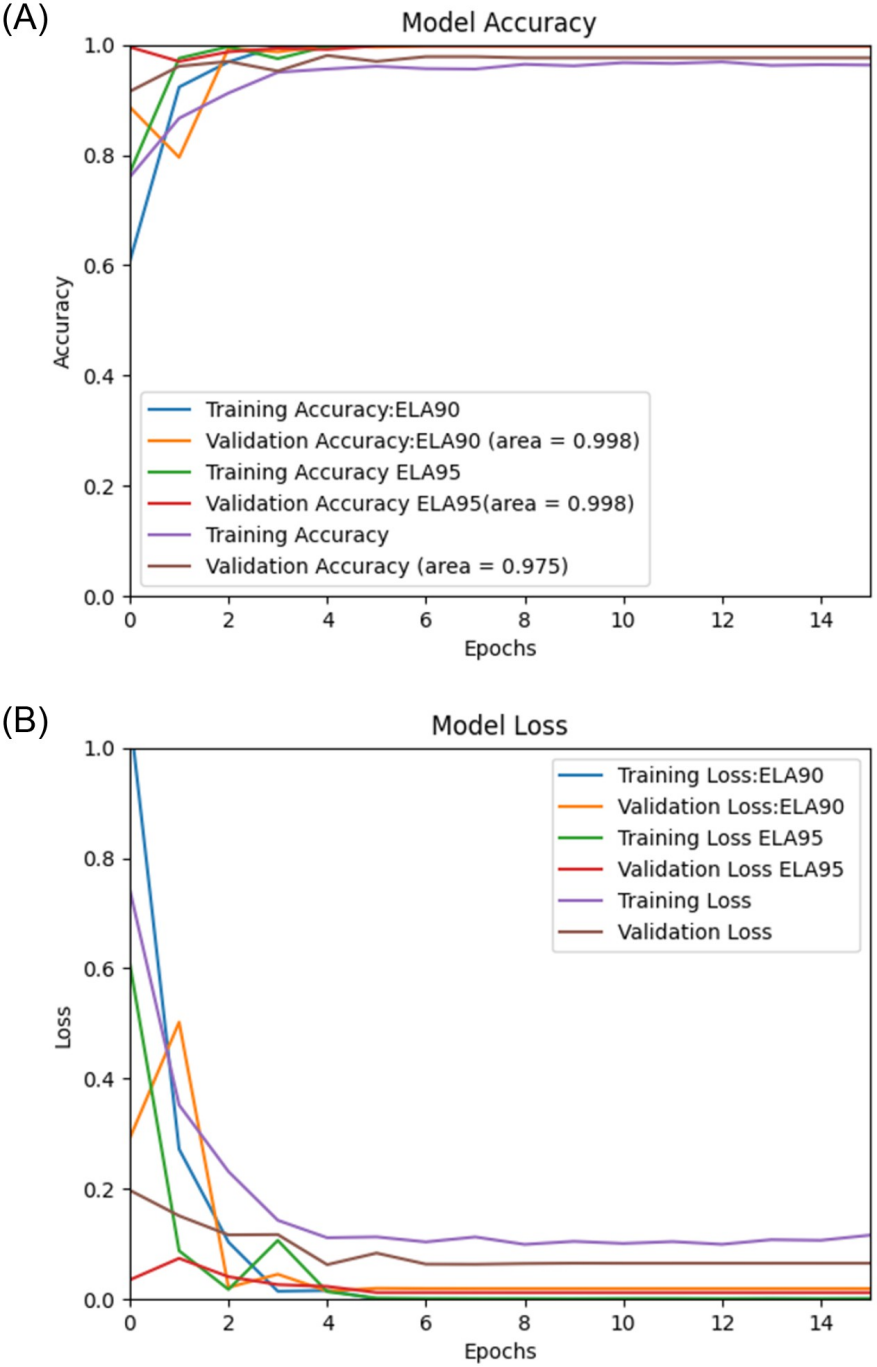
**(A,B):** Validation Loss and ROC curve on Improved Dataset [36].

**Fig 7:**
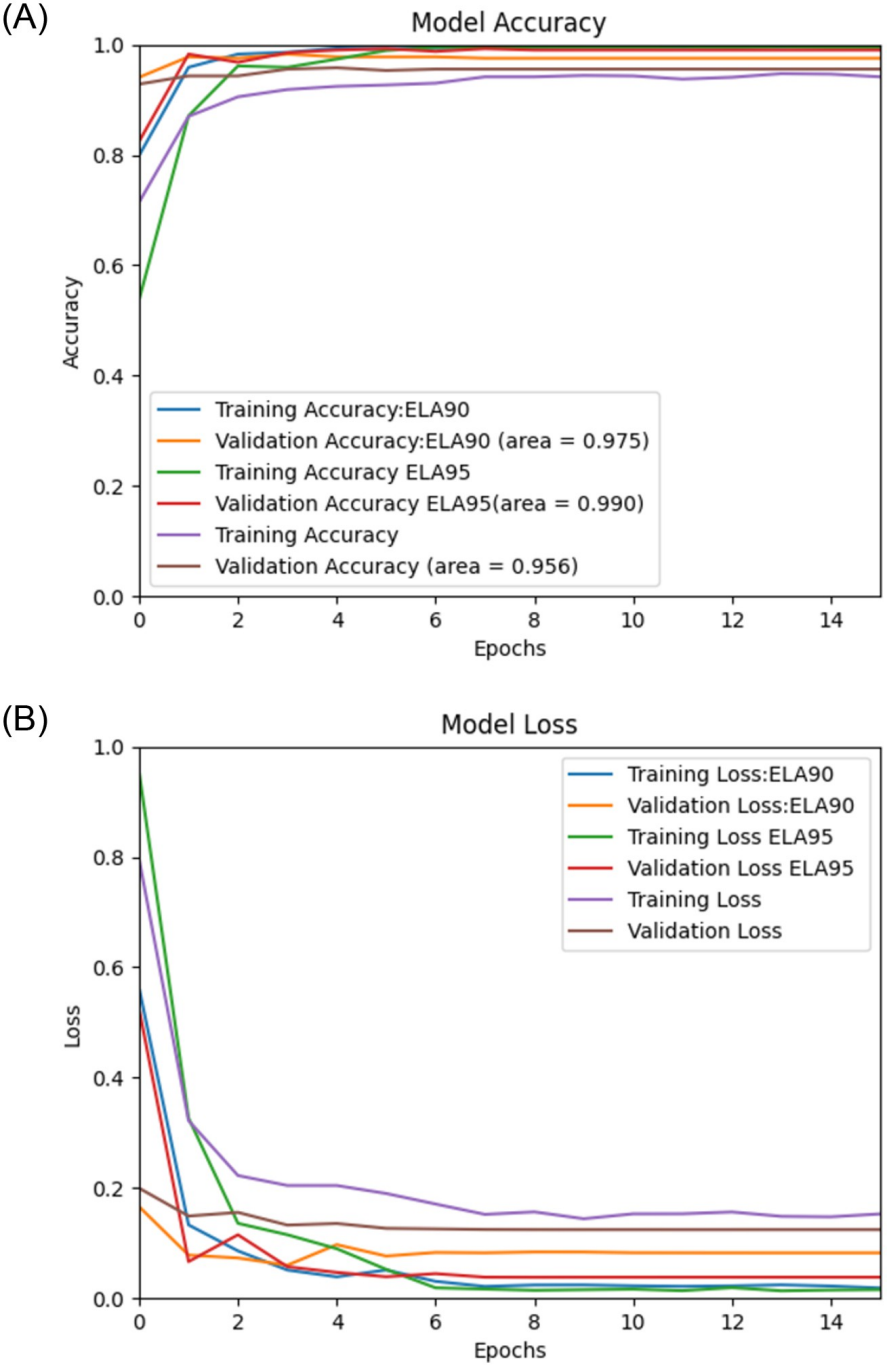
**(A,B):** Validation Loss and ROC curve on Challenge-A Dataset [37].

**Fig 8:**
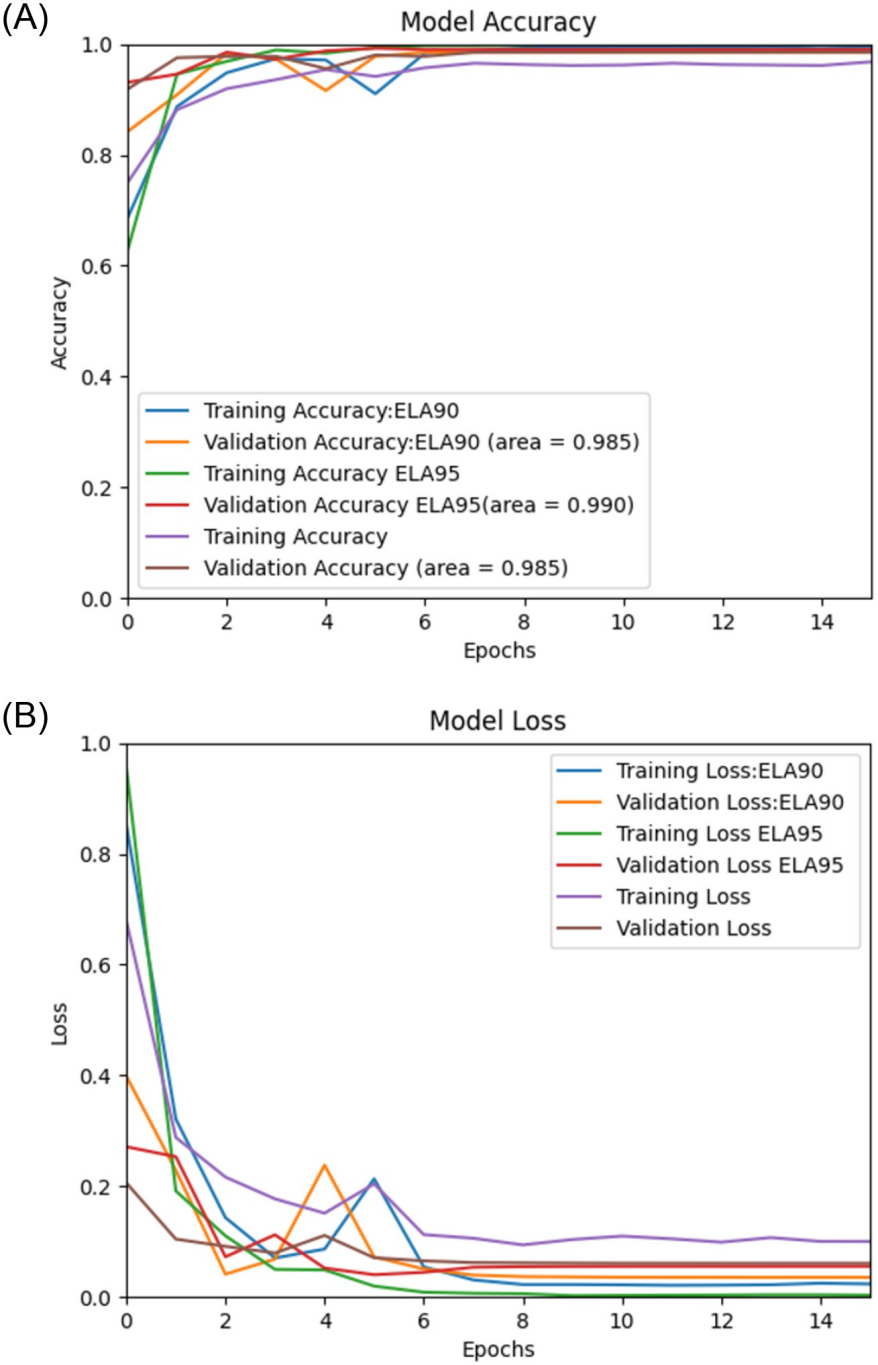
**(A,B):** Validation Loss and ROC curve on Challenge-B Dataset [37].

**Fig 9:**
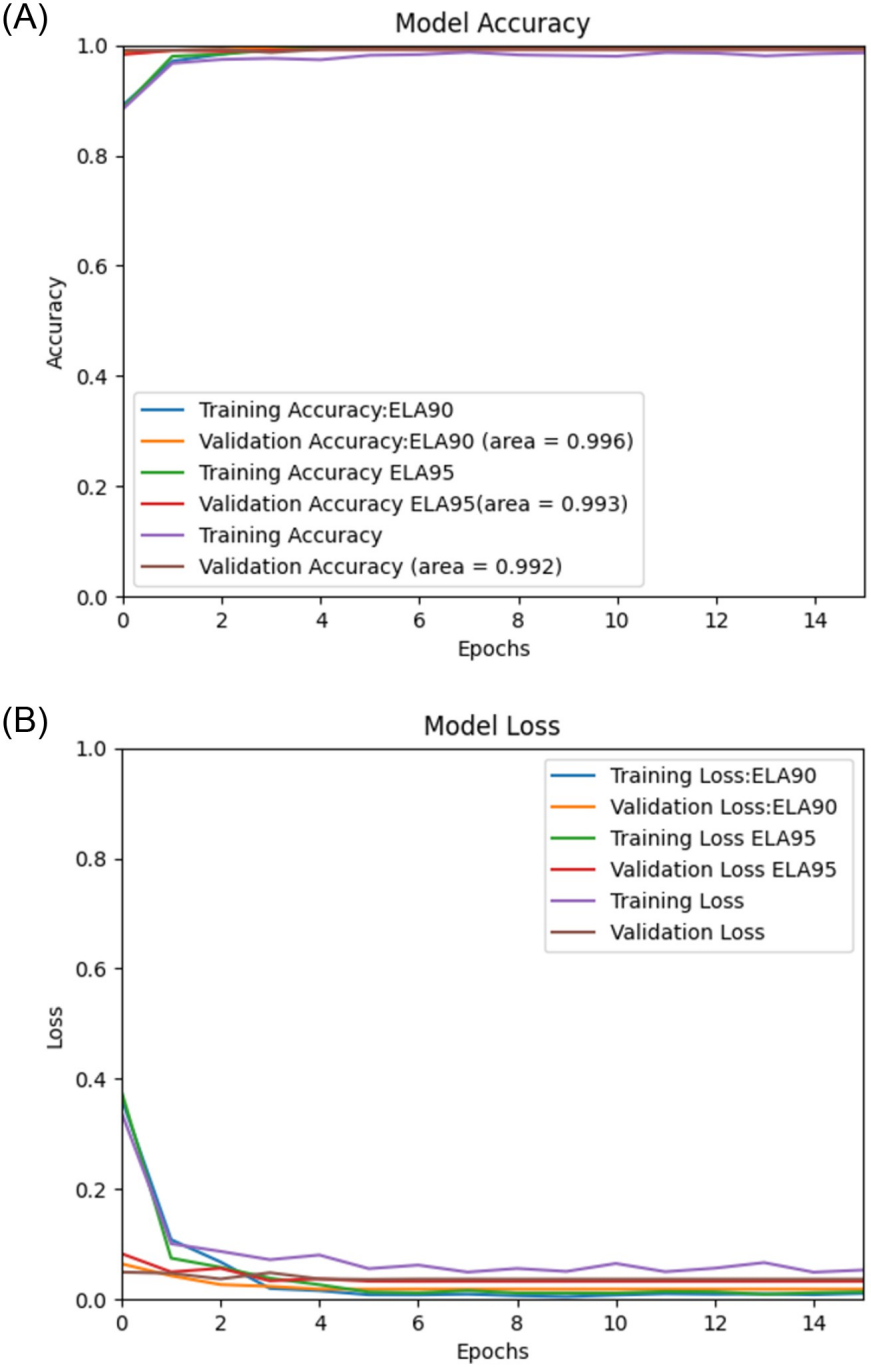
**(A,B):** Validation Loss and ROC curve on Dredze Dataset [17].

**Fig 10:**
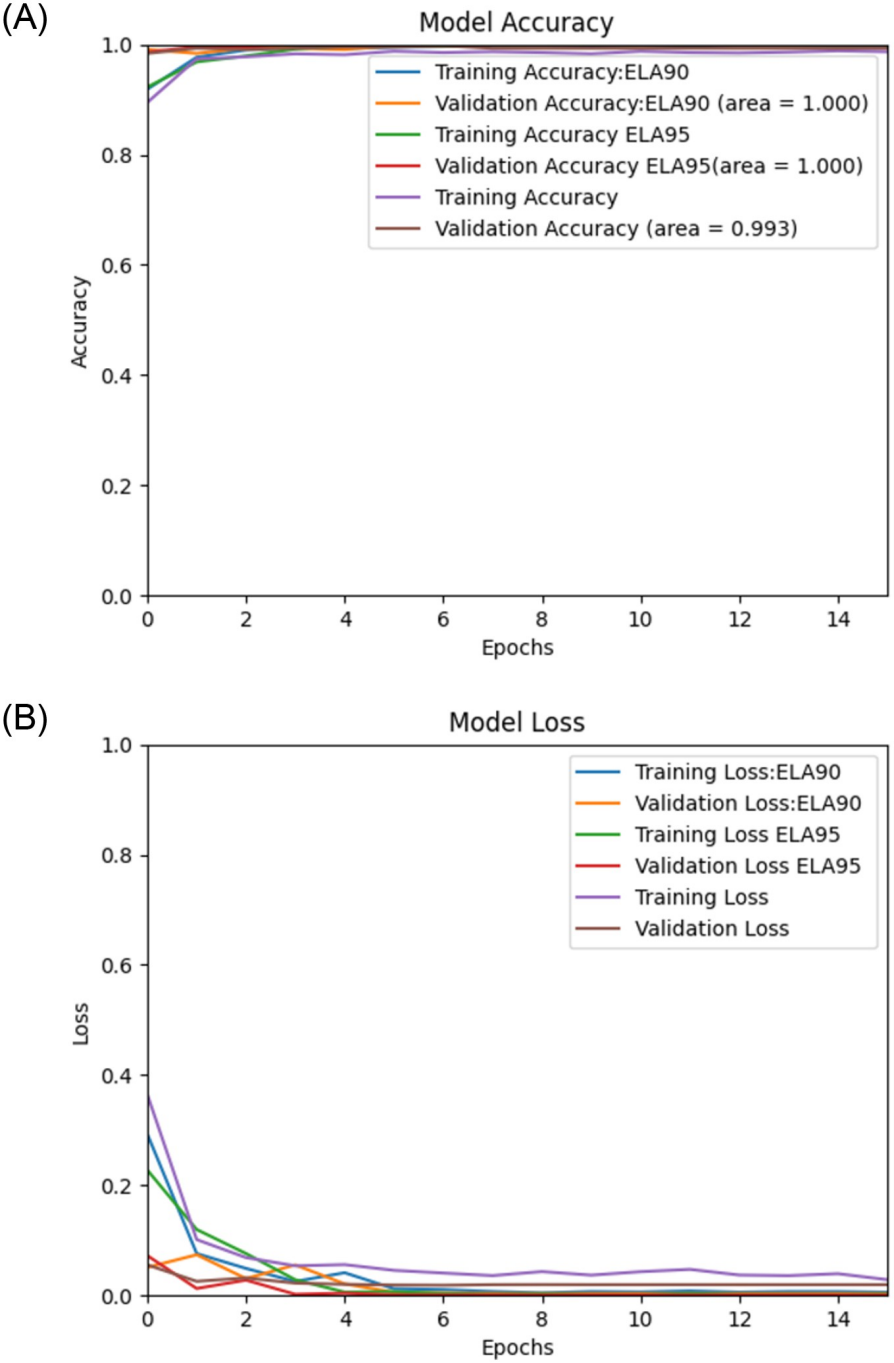
**(A,B):** Validation Loss and ROC curve on ISH Dataset [18].

Using the proposed Deep Learning model along with the pre-processing, we performed experiments on image spam datasets as mentioned earlier, namely, “Improved” [36], Challenge-A [37], Challenge-B [37], Dredze [17], and ISH [18], and the results of the experiments are then validated by using the validation sets. The (Figs 6–10) presents the ROC curve and the validation loss of different experiment, on the datasets.

From the above, we can see that the proposed model with ELA pre-processing achieved a higher accuracy than the non ELA images in all the datasets. We also note that there is insignificant difference in the accuracy for the two compression ratios used in generating the ELA images i.e., 90% and 95%.

The computational speeds of the proposed CNN model along with the speed of execution are shown in Table 5.

**Table 5:** Computational Speeds of model.

Base Model Name	Speed/epoch	Speed/Step	Number of Parameters
BiT-M R50x1	7s	337ms	23500352

**Fig 11:**
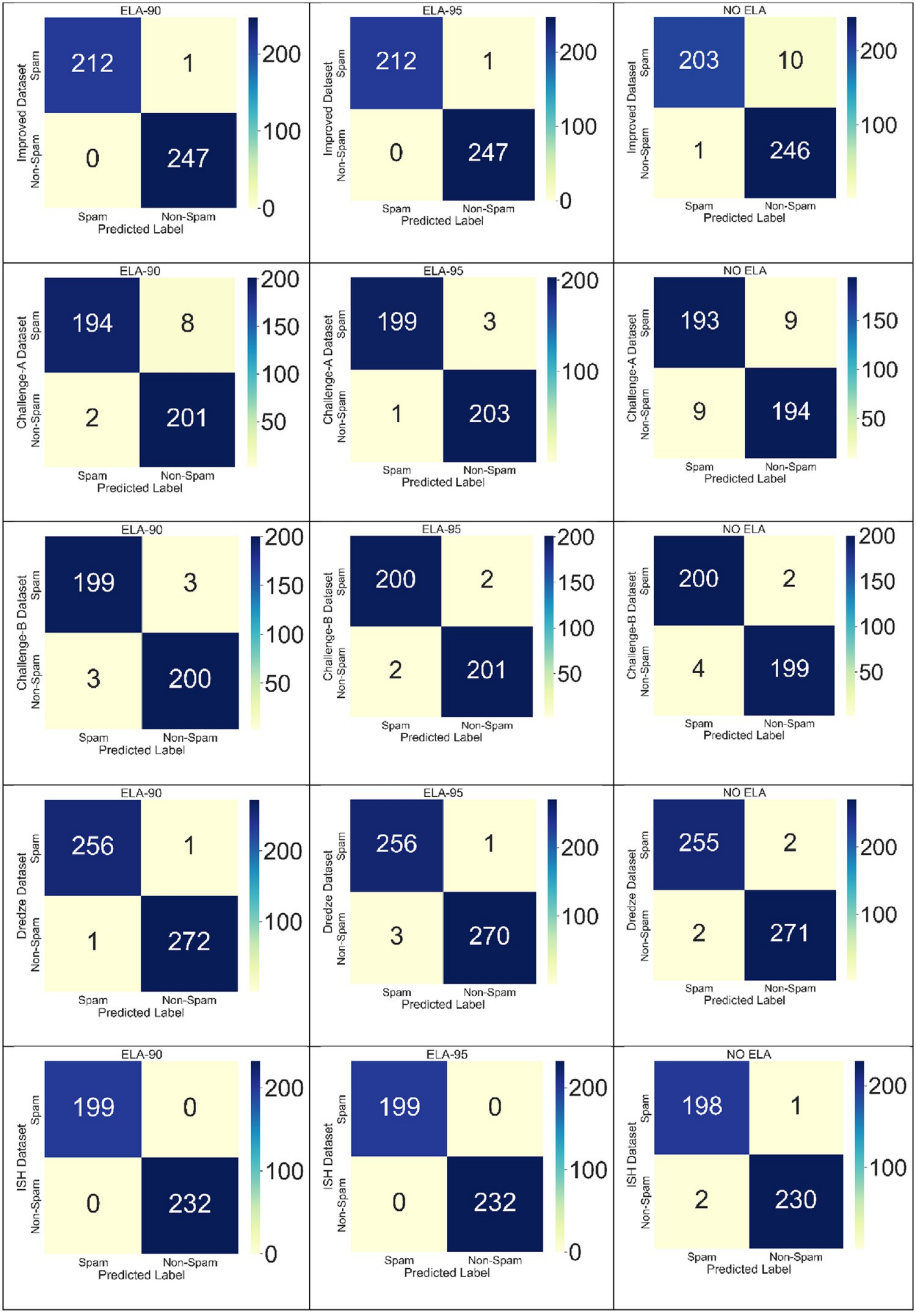
Resulting confusion matrix.

The confusion matrix for the various experiment on different datasets with compression ratio of 90%, 95% and Non-ELA are shown in Fig 11.

The model performance measures namely the accuracy, precision, recall and f1-score for the various experiment on different datasets are shown in Table 6.

**Table 6:** Model accuracy, precision, recall and f1-score.

	CNN Model	ACC	PRECISION	RECALL	F1-SCORE
**Improved Dataset [36]**	ELA-90	**99.78**	100	99.53	99.76
	ELA-95	**99.78**	100	99.53	99.76
	NO ELA	97.61	95.51	99.31	97.36
**Challenge-A Dataset [37]**	ELA-90	97.53	98.98	96.04	97.49
	ELA-95	**99.01**	99.50	98.51	99.00
	NO ELA	95.56	95.54	95.54	95.54
**Challenge-B Dataset [37]**	ELA-90	98.52	98.51	98.51	98.51
	ELA-95	**99.01**	99.01	99.01	99.01
	NO ELA	98.52	98.04	99.01	98.52
**Dredze Dataset [17]**	ELA-90	**99.62**	99.61	99.91	99.61
	ELA-95	99.25	98.84	99.61	99.22
	NO ELA	99.25	99.22	99.22	99.22
**ISH Dataset [18]**	ELA-90	**100**	100	100	100
	ELA-95	**100**	100	100	100
	NO ELA	99.30	99.00	99.50	99.25

Our proposed CNN model performed very well on other publicly available datasets as given in Table 7 and achieved tremendous improvement in the accuracies as compared with the other approaches.

**Table 7:** Comparison of accuracy obtained for Improved, Challenge, Dredze and ISH Datasets.

	Dataset-3 Improved	Dataset-4 A Challenge	Dataset-4 B Challenge	Dataset-1 Dredze	Dataset-2 ISH
Proposed Model (ELA-90)	**99.78**	97.53	98.52	**99.62**	**100**
Proposed Model (ELA-95)	**99.78**	**99.01**	**99.01**	99.25	**100**
Proposed Model (NO ELA)	97.61	95.56	98.52	99.25	99.25
A.Annadatha et al [36]	70.00	-	-	-	97.00
Aneri. et al [37]	-	69.32	69.32	98.00	97.00
Sriram. S et al [38]	97.30	-	-	97.30	99.80
Aaisha et al [16]	-	-	-	-	96.00

## 6. Conclusion

Image spam detection is a binary classification problem that uses models to extract and train features for image classification. Models that rely on manual feature extraction are not suitable when presented with specially handcrafted image spam datasets. However, models that automatically extract image features from input images, such as those based on convolutional neural networks (CNNs), perform extremely well even on challenge datasets. The performance of such models can be improved by fine-tuning the hyper-parameters of the model and pre-processing the input images.

In our experiment, we used a deep learning model based on CNN and transfer learning to extract image features from input images that had been pre-processed using error level analysis (ELA). Our proposed approach achieved extremely high levels of accuracy not only on standard image spam datasets, but also on improved and challenge datasets. With the use of ELA, we were able to increase the efficiency and reduce the computational costs of the training process.

In our future work, we hope to apply the proposed approach to other image datasets using automatic parameter tuning methods and perform various statistical analysis on the performance.
